# Predictive Feedback and Conscious Visual Experience

**DOI:** 10.3389/fpsyg.2012.00620

**Published:** 2013-01-21

**Authors:** Matthew F. Panichello, Olivia S. Cheung, Moshe Bar

**Affiliations:** ^1^Athinoula A. Martinos Center for Biomedical Imaging, Massachusetts General Hospital and Harvard Medical SchoolCharlestown, MA, USA; ^2^Gonda Multidisciplinary Brain Research Center, Bar-Ilan UniversityRamat-Gan, Israel

**Keywords:** predictions, context, object recognition, priming, visual awareness, top-down, perception, associative processing

## Abstract

The human brain continuously generates predictions about the environment based on learned regularities in the world. These predictions actively and efficiently facilitate the interpretation of incoming sensory information. We review evidence that, as a result of this facilitation, predictions directly influence conscious experience. Specifically, we propose that predictions enable rapid generation of conscious percepts and bias the contents of awareness in situations of uncertainty. The possible neural mechanisms underlying this facilitation are discussed.

## Introduction

Perception has evolved to transform raw sensory signals into information that can guide behavior. But perception is not a purely stimulus-driven phenomenon; the brain perceives the world proactively. Endogenous influences, such as attention, memory, and mood, guide perception to ensure that informative representations of the environment are generated as efficiently as possible.

In this review, we focus on the influence of predictions on visual perception. The predictions we discuss are not analogous to the deliberate, elaborative foresight that individuals may engage in when planning for the future (e.g., thinking about which route home from work will have the least traffic). Instead, we use the term “prediction” here to refer to expectations about the immediate sensory environment based on previous experience and learning. Through everyday experience, individuals learn many types of regularities in the world, such as associations among items or events. For example, a yellow traffic light is almost always followed by a red light. Insofar as the environment is generally regular, it is, to an extent, predictable. Expectations about the environment can be derived from these learned regularities and used to guide sensory processing, presumably via top-down (descending) projections linking brain regions involved in generating expectations with lower-level sensory regions.

If predictions guide processing in visual areas, it follows that this facilitation may have consequences for conscious perception^1^. We argue that predictive mechanisms may shape the contents of visual awareness during instances of sensory ambiguity, allowing subjective experience of the world to remain informative and coherent. Additionally, when sensory input is less ambiguous, predictions may allow percepts to be generated more quickly and with less interference by sensory noise. In other words, the expectation elicited by a yellow traffic light may cause a driver to consciously perceive the subsequent red light more quickly than otherwise possible. We conclude by discussing the neural mechanisms underlying these predictive influences.

## Predictions Influence Conscious Perception

### Predictions guide the interpretation of ambiguous stimuli

One method for studying the influence of predictions on awareness is to show observers stimuli with multiple perceptual interpretations and determine if predictions affect what they see. The popular perceptual phenomenon known as binocular rivalry provides a convenient means to accomplish this. Binocular rivalry occurs when a unique image is presented to each eye; interpretation of the stimuli is inherently ambiguous because the two eyes provide conflicting information about the shared portion of the visual field. The brain must work out the most likely interpretation of this impossible input (Hohwy et al., 2008). The resolution of this problem is striking: the two stimuli do not fuse together into a blended percept, but rather alternately dominate perception (for reviews of binocular rivalry and multistable perception more generally, see Leopold and Logothetis, 1999; Blake and Logothetis, 2002; Sterzer et al., 2009).

**Figure 1:**
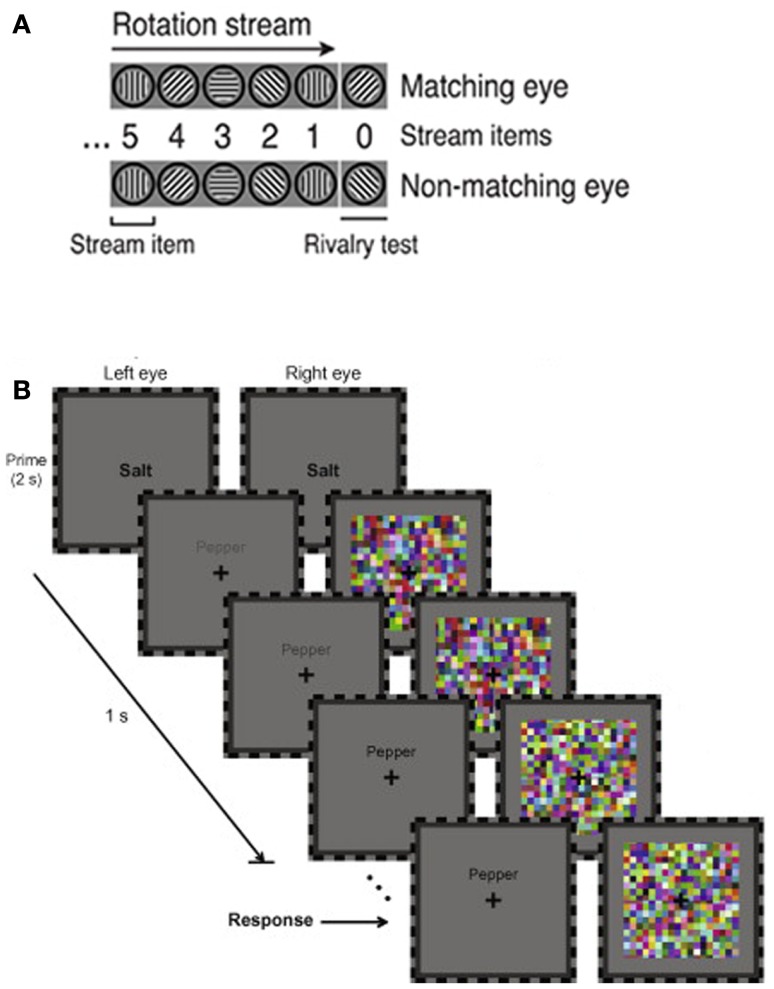
**Predictions affect the outcome of binocular rivalry**. **(A)** When a stream of images depicting a grating rotating in 45° increments is presented to both eyes, observers are more likely to perceive the grating consistent with this pattern (the “matching eye” stimulus) at the onset of rivalry. Adapted, under creative commons license, from Denison et al. (2011). **(B)** A word prime (“salt”) causes a semantically related target word (“pepper”; left eye) to break suppression by dynamic noise (right eye) during rivalry more quickly than when the prime and target are not related. The increasing contrast of the target over time helps ensure that the target eventually breaks suppression. Adapted, with permission, from Costello et al. (2009).

Binocular rivalry is sensitive to many factors, including low-level stimulus properties (e.g., Kaplan and Metlay, 1964; Fahle, 1982) and higher-order endogenous influences such as attention, imagery, and affect (e.g., Ooi and He, 1999; Pearson et al., 2008; Anderson et al., 2011), so a single process or mechanism should not be credited as the “source” of rivalry (Blake and Logothetis, 2002). However, recent findings suggest that predictions play a role in resolving the ambiguity inherent in this phenomenon. For example, a stimulus will tend to dominate rivalry if it has been presented more frequently than the competing stimulus in the recent past; the likelihood of each stimulus is estimated based on recent experience, generating expectations that can guide perception (Chopin and Mamassian, 2012). The tendency of stimuli to change position smoothly during motion can also provide predictive cues for perception. Thus, observers who view a stream of images depicting a rotating grating are more likely to perceive the stimulus consistent with the rotation trajectory at the onset of rivalry (Figure 1A; Denison et al., 2011). Verbal stimuli can also produce an expectation for semantically related stimuli. To demonstrate this, Costello et al. (2009) used a variant of binocular rivalry, termed continuous flash suppression, which relies on the tendency of high-contrast dynamic noise to dominate awareness when presented in rivalry with another stimulus (Tsuchiya and Koch, 2005). Costello et al. found that a word presented to one eye breaks suppression by high frequency noise presented to the other eye more quickly when a semantically related prime is displayed prior to rivalry (Figure 1B).

**Figure 2:**
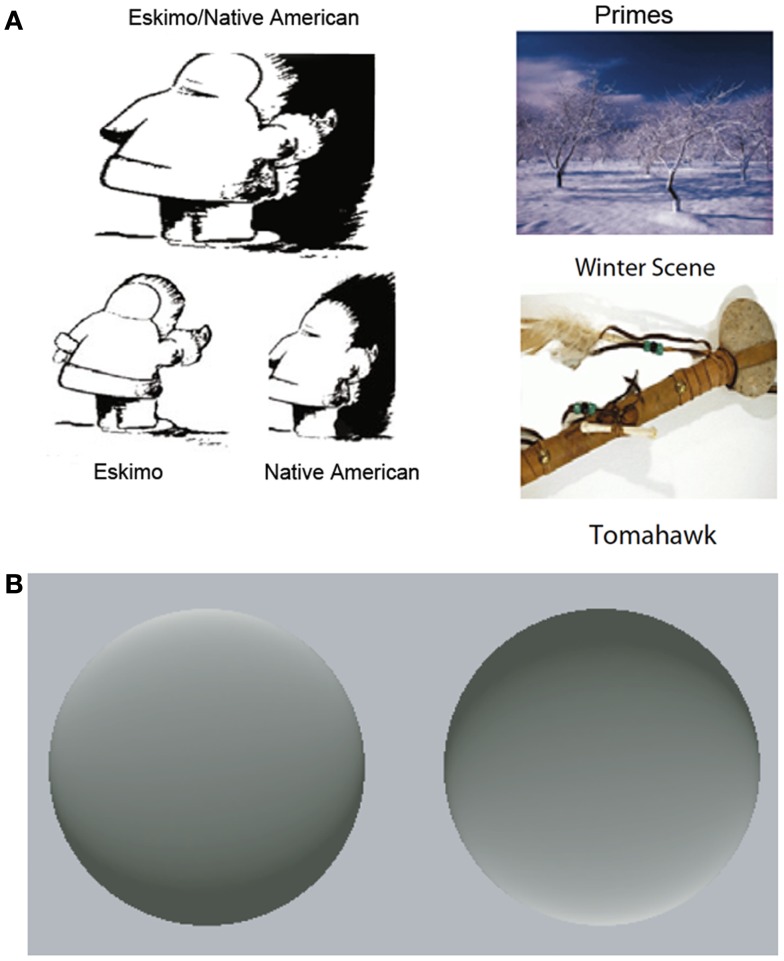
**Predictions affect the interpretation of ambiguous stimuli**. **(A)** Example of an ambiguous figure with its two possible perceptual interpretations emphasized (left). Viewing a prime related to one of the interpretations (right) biases perception in favor of that interpretation. Adapted, with permission, from Goolkasian and Woodberry (2010). Copyright ©by Springer Publishing. **(B)** These two disks appear to be concave and convex, respectively, due to learned assumption that light comes from above. Experience violating this assumption causes a new shading scheme to maximally evoke the illusion of contour. Adapted from Adams et al. (2004). Copyright ©by Nature Publishing Group.

In addition to binocular rivalry, bistable figures reveal the ability of top-down predictions to influence conscious perception. Bistable figures have two mutually exclusive interpretations that alternately dominate awareness during viewing. However, a predictive cue related to one of the interpretations of a bistable figure can bias perception in favor of that interpretation (Figure 2A; Bugelski and Alampay, 1961; Balcetis and Dale, 2007; Goolkasian and Woodberry, 2010). Stimuli can also display bistable motion; an array of moving dot stimuli can be arranged so that observers perceive a rotating cylinder with spontaneous reversals in rotation direction. However, when observers are led to expect that the cylinder will consistently rotate in a particular direction, this interpretation dominates perception (Sterzer et al., 2008).

Intriguingly, predictions can alter the perception of stimuli that are normally perceptually stable. For example, most individuals see the left disk in Figure 2B as a convex and the right disk as concave. However, the contour of each disk is actually ambiguous: this subjective interpretation is driven by the shading of the figures and the learned assumption that “light comes from above” (Brewster, 1826; Sun and Perona, 1998; Adams et al., 2004). New experience contradicting this assumption can cause a substantial shift in inferred light position; as a result, a new shading scheme will maximally evoke the illusion of contour (Adams et al., 2004). Similarly, individuals learn over time that fast-moving objects are less common than slow moving and stationary objects, producing an expectation that can guide perception. New visual experience dominated by fast-moving stimuli alters this expectation, and by extension, subsequent motion perception (Stocker and Simoncelli, 2006; Sotiropoulos et al., 2011). Together, these studies demonstrate that predictions derived from experience can affect how observers see the world.

### Predictions facilitate perception during object recognition

Even when visual input is not ambiguous, predictive mechanisms may influence conscious perception. We hypothesize that predictions allow conscious percepts to be generated more efficiently and with less inhibition by sensory noise^2^. The most direct evidence for this claim is provided by studies showing that predictions can facilitate perception of objects when visual input is noisy. Observers viewing fragmented object figures are more likely to perceive the objects when they are informed about their category (e.g., “an animal” for an elephant; Reynolds, 1985). Similarly, observers instructed to indicate their subjective visual experience report successfully perceiving expected stimuli at greater levels of degradation or lower contrast than unexpected stimuli (Eger et al., 2007; Esterman and Yantis, 2010; Melloni et al., 2011). In the latter study, electrophysiological activity evoked by expected stimuli correlated with subjective experience earlier than that evoked by unexpected stimuli, suggesting that predictions reduced the latency of neural activity related to conscious perception (Melloni et al., 2011).

A large body of research demonstrates that predictions make object recognition faster and more accurate. These studies provide additional, although indirect, evidence for predictive facilitation of conscious perception. These results must be interpreted with caution for two reasons. First, object recognition is a multifaceted process, with multiple stages that may be amenable to influence by predictive mechanisms (e.g., the assignment of semantic attributes to the perceived object). Second, most of the studies cited below examined the influence of predictions on behavioral proxies of recognition such as reaction time rather than subjective reports of perception. However, it will be worthwhile for future research to determine if predictive facilitation of object recognition is due, at least in part, to faster and more accurate generation of object percepts.

For instance, contextual facilitation of object recognition arises from the knowledge that certain objects often reliably co-occur in particular settings. Cubicles and copy machines, but not octopuses and cars, are found inside office buildings. Furthermore, objects within a context are often arranged in a regular manner: computer monitors rest on desks inside of cubicles. Over time, observers learn many sets of such regularities, termed schemata or “context frames” (Friedman, 1979). Cues within a scene can activate associated context frames, allowing observers to predict other features of the environment (Bar, 2004). Accordingly, observers identify objects faster and more accurately when they are shown in their typical environment (e.g., a toaster in the kitchen, Biederman, 1972; Palmer, 1975; Biederman et al., 1982; Davenport and Potter, 2004) or are preceded by an object drawn from the same context (e.g., a bedroom dresser and a vanity mirror, Gronau et al., 2008; see also Sachs et al., 2011). Conversely, recognition is impaired when the expected spatial relationships among objects in a scene are disrupted (Biederman, 1972).

Associations among stimuli can be reinforced over a lifetime of experience. However, they can also be generated quite quickly under artificial conditions in the laboratory (e.g., Chun and Jiang, 1998, 1999; Aminoff et al., 2007; Kim et al., 2009; den Ouden et al., 2010; Turk-Browne et al., 2010). Thus, associative learning appears to be quite flexible and continuously updated. For instance, in a study of perceptual prediction (den Ouden et al., 2010), observers were asked to judge whether a degraded image depicted a face or a house. An auditory cue presented at the start of each trial signaled which stimulus type was more likely to appear, and the experimenters manipulated the predictive strength of these cues over time. Modeling of the behavioral data demonstrated that observers updated their estimates of stimulus category likelihood on a trial-by-trial basis and responded more quickly when cues were highly predictive.

**Figure 3:**
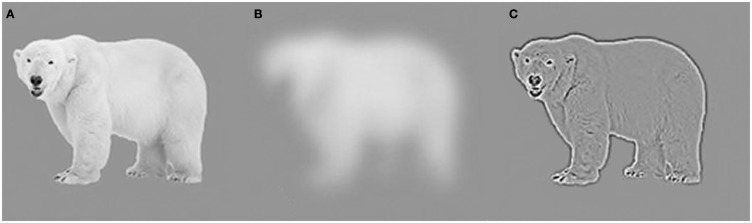
**An image filtered to include (A) both low and high spatial frequency information (B) predominantly low spatial frequencies (C) predominantly high spatial frequencies**. The low spatial frequency information in (B) is not sufficient to identify the polar bear but nevertheless substantially constrains the possible interpretations of the stimulus.

Apart from learned associations among stimuli, preliminary processing of visual information can also create expectancies that facilitate perception. Visual stimuli contain information distributed across a range of spatial frequencies (Figure 3A). Low spatial frequency (LSF) information is rapidly extracted from incoming sensory input and encodes gross properties such as the global shape of the environment and its constituent objects (Figure 3B). In contrast, high spatial frequency (HSF) information is processed more slowly and corresponds to edges and fine details (Figure 3C; Shapley, 1990; Schyns and Oliva, 1994; Bar et al., 2006). It is possible that LSF information can elicit predictions (Bar, 2003) because objects in the same basic-level category often display a similar global shape (Rosch et al., 1976). For example, individual dogs vary tremendously, but all dogs share roughly the same gross features (e.g., four legs and a tail), which immediately differentiate them from members of many other basic-level categories. With experience, we come to learn the defining features of many kinds of objects. Thus, while LSF information lacks fine detail, it is sufficient to trigger general category information stored in memory that guides interpretation of the stimulus (Bar, 2003; Bar et al., 2006; Oliva and Torralba, 2007). In addition to individual objects, different exemplars of basic-level scenes (such as a city street) also share global features that can constrain and facilitate recognition (Bar, 2004; Oliva and Torralba, 2007). Thus, rapid processing of LSF information may explain the remarkable ability of observers to extract the “gist” of a scene at a glance (Biederman et al., 1974; Thorpe et al., 1996; Oliva and Torralba, 2007).

To summarize, predictions may shape the contents of awareness when visual input is ambiguous and enable faster and more sensitive conscious perception under less strenuous circumstances. The ability of predictions to shape perception under conditions of uncertainty is clearly advantageous; it is better to generate a meaningful interpretation of the world, informed by previous experience, than to faithfully represent a noisy sensory signal. When visual input is more informative, predictions still guide sensory processing to make conscious perception as efficient as possible. Note that although we suggest that predictions influence conscious experience, predictions themselves may be learned and applied without awareness (e.g., Chun and Jiang, 1998, 1999; Kim et al., 2009; Turk-Browne et al., 2010). We next consider how, at the neural level, predictions may guide perception.

## The Neural Basis of Prediction

### Descending cortico–cortico projections allow predictions to guide sensory processing

An increasingly popular framework (e.g., Mumford, 1992; Rao and Ballard, 1999; Friston, 2005; Friston and Kiebel, 2009), termed “predictive coding”, posits that top-down predictions facilitate perception by reducing the need to reconstruct the environment via exhaustive bottom-up analysis of incoming sensory information. Validated predictions efficiently explain away some of the sensory input, conserving resources for analysis of unpredicted components. The hierarchical organization of sensory cortex suggests a natural computational architecture for the integration of top-down predictions and bottom-up sensory information. Ascending projections in sensory cortex are sparse and focused, terminating in predominately layer 4 of cortical targets, whereas descending connections project to a larger number of region and innervate their targets in superficial and deep layers of cortex (Felleman and Van Essen, 1991; Friston, 2009). Predictive coding proposes that descending projections convey predictions about the content or organization of the sensory input. Ascending projections convey any information incongruent with the predictions (i.e., an “error term”) received from higher sensory areas. Thus, reciprocally connected cortical areas are able to engage in a dynamic process in which predictions are modified based on incoming sensory input until the higher-level region is able to arrive at reasonable approximation of the incoming input (Ullman, 1995; Friston, 2005).

This predictive coding framework has traditionally been presented with the hierarchical organization of sensory cortex in mind (e.g., Mumford, 1992; Friston, 2005). However, the existence of reciprocal connections between prefrontal and visual cortices in the macaque (Webster et al., 1994; Cavada et al., 2000) and evidence of functional interaction between prefrontal and inferior temporal regions in humans (e.g., Bar et al., 2006; Kveraga et al., 2007; Axmacher et al., 2008) are well documented. These findings suggest that prefrontal regions provide an additional source of feedback to the visual hierarchy (but note that prefrontal regions do not seem to be arranged hierarchically with respect to each other; Yeo et al., 2011). Accordingly, expectation-based prefrontal modulation of sensory processing has been reported (Bar et al., 2006; Summerfield et al., 2006; Eger et al., 2007; Kveraga et al., 2007; Gamond et al., 2011). Predictive mechanisms may thus be instantiated in a variety of brain regions, but consistently seem to depend on the use of descending connections to allow the dynamic comparison of predictions with sensory input.

Converging imaging and computational evidence supports the proposed role of predictive feedback during perception. If descending predictions efficiently facilitate the interpretation of sensory input, then predicted stimuli should evoke less activity in sensory cortex, consistent with the notion that stimulus-driven activity conveys an “error term” communicating the remainder of the signal that has not been explained by top-down predictions. This effect should be especially pronounced in visual processing regions that are particularly devoted to the processing of the predicted stimuli. Accordingly, shapes that appear in an expected location in the visual field elicit less activity in the retinotopic region V1 (Alink et al., 2010), and expected face and place stimuli elicit less activity in face and place-sensitive regions of high-level visual cortex (Egner et al., 2010).

Moreover, because predictive feedback from higher-level processing regions shapes the activity of lower-level sensory regions, activity in lower-level sensory regions should track surprisingly sophisticated aspects of the sensory input. A subset of retinotopic neurons in visual cortex seem sensitive to stimuli outside of their receptive fields, a property replicated in a computational model of visual cortex allowing for predictive feedback to these cells (Rao and Ballard, 1999). Similarly, activity in low-level retinotopic visual areas varies with image recognition (Hsieh et al., 2010; see also Gorlin et al., 2012), conscious experience of figure-ground segregation (Lamme et al., 1998, 2002), and target detection (Supèr et al., 2001), implicating top-down modulation by higher-order areas.

To summarize, expectations derived from learned regularities are able to guide perception via descending feedback targeting sensory regions. We next elaborate on these ideas by reviewing the neural mechanisms for two previously discussed predictive processes: predictions based on contextual associations and predictions based on preliminary LSF information extracted from stimuli. Different neural generators may support these mechanisms, but evidence suggests that both may require interaction between higher cortical areas and visual cortex to allow top-down predictions to shape sensory processing.

### Predictions based on contextual associations among objects

**Figure 4:**
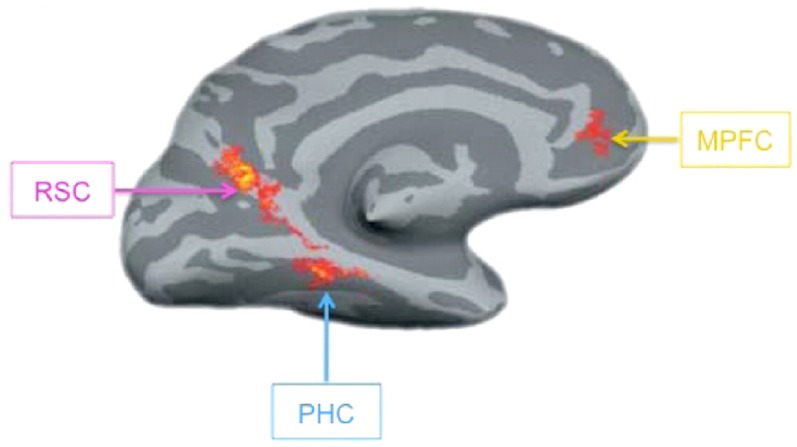
**The context network**. A contrast of strong and weak context object stimuli, revealing the context network on the left hemisphere of an inflated cortical surface. RSC, retrosplenial complex; PHC, parahippocampal cortex; MPFC, medial prefrontal cortex. Reprinted from Kveraga et al. (2011). Copyright ©by National Academy of Sciences/Highwire Press.

In the previous section, we have suggested that contextual associations among objects can create expectancies that guide perception. A distributed network including parahippocampal cortex (PHC), retrosplenial complex (RSC), and medial prefrontal cortex (mPFC) mediates these contextual associations (Figure 4; Bar and Aminoff, 2003; Kveraga et al., 2011). To localize this context network, activity elicited by objects that are strongly associated with a particular context is compared with that elicited by objects that are only weakly associated with any particular context. For example, golf carts are usually found in the context of a golf course and are thus closely associated with other objects that share this context such as golf clubs and golf balls, while cameras lack strong associations because they are found in a variety of contexts and thus do not consistently appear with any particular set of objects (Bar and Aminoff, 2003; stimuli available at http://barlab.mgh.harvard.edu/ContextLocalizer.htm). Thus, associative processing seems to specifically engage these context network regions. Further support for this claim is provided by the fact that these regions are recruited in a variety of tasks that call on contextual associations, such as memory encoding (Peters et al., 2009), navigation (Rauchs et al., 2008; Brown et al., 2010), and future thought (Szpunar et al., 2009).

There are several mechanisms by which contextual processing may modulate activity in regions related to visual perception. PHC itself is situated in the ventral visual stream and lesions of this region in the monkey severely impair object recognition (Buckley and Gaffan, 1998; Murray and Mishkin, 1998). Thus, one possibility is that other context network regions facilitate the emergence of situation-specific representations in PHC in a top-down manner (Bar, 2007; Bar et al., 2008). MEG recordings have shown that strong context objects elicit phase-locking (a measure of functional interaction) between PHC and other context network regions as early as 170 ms after stimulus onset (Kveraga et al., 2011), suggesting that the interaction between these regions can occur early enough to influence perception. Alternatively, context network regions may also influence processing in lateral occipital cortex (LOC), a region implicated in object recognition (e.g., Malach et al., 1995; Grill-Spector et al., 2001). Contextual information modulates LOC response during recognition (Altmann et al., 2004; Gronau et al., 2008; MacEvoy and Epstein, 2011), suggesting that this region may be receiving feedback signals from contextual processing regions. Further research will need to clarify the precise neural mechanism by which contextual predictions guide processing in sensory cortex.

### Predictions based on low spatial frequency information

The prefrontal cortex, specifically orbitofrontal cortex (OFC), may play a critical role in generating predictions based on LSF information (Bar, 2003; Bar et al., 2006). In this proposed framework, LSF information is extracted from visual input and projected to OFC via the magnocellular cells of the dorsal visual stream, which preferentially respond to and rapidly conduct LSF information (Maunsell et al., 1990; Shapley, 1990; Merigan and Maunsell, 1993; Bullier and Nowak, 1995; Chen et al., 2007). As we have suggested, the coarse representations conveyed by LSFs are sufficient to activate a subset of possible candidates regarding the identity of the visual input (Bar, 2003). These predictions are then projected back to object recognition regions in inferior temporal cortex, facilitating perception (Bar et al., 2006).

Consistent with this proposal, LSF images evoke activity in OFC prior to inferior temporal areas; thus, LSF information reaches prefrontal cortex quickly enough to influence recognition processes in the ventral visual stream (Bar et al., 2006). Furthermore, LSF images elicit significant functional coupling between early visual areas and OFC and between OFC and ventral stream areas (peaking approximately 85 and 135 ms post-stimulus, respectively) while HSF images do not (Bar et al., 2006). These findings are consistent with the rapid transmission of LSF information to OFC, followed by top-down feedback from OFC to the ventral stream.

Facilitatory feedback originating in OFC has also been shown using stimuli designed to preferentially stimulate the dorsal magnocellular pathway (Kveraga et al., 2007). Magnocellular cells are sensitive to small differences in luminance contrast but are insensitive to color, whereas the parvocellular cells known to dominate the ventral stream are sensitive to color but relatively insensitive to luminance (Livingstone and Hubel, 1988). Accordingly, line drawings of objects in which figure and ground are identical in color but slightly different in luminance preferentially stimulate magnocellular cells (i.e., “M-biased” stimuli; Steinman and Steinman, 1997; Cheng et al., 2004; Kveraga et al., 2007). Such stimuli can be used to examine whether the magnocellular pathway is indeed important for conveying information to OFC, enabling top-down facilitation. Indeed, M-biased stimuli preferentially stimulate OFC and elicit functional interaction between early visual areas, OFC, and inferior temporal cortex (Kveraga et al., 2007). Furthermore, the degree of OFC activation elicited by M-biased stimuli is inversely correlated with reaction time for object recognition, suggesting that the processing occurring in OFC is indeed facilitating perception (Kveraga et al., 2007). Intriguingly, despite this facilitation, M-biased stimuli elicited less activity in ventral visual regions than stimuli designed to stimulate parvocellular cells, providing indirect evidence that predictive feedback may reduce the need for exhaustive bottom-up processing during recognition.

We have suggested that LSF representations in OFC trigger associations with object and category information stored in memory, which then serve as predictions that guide sensory processing. To activate memory representations, OFC should interact with hippocampal regions in the medial temporal lobe, known to support long-term memory (for a review, see Squire et al., 2004). Indeed, OFC and medial temporal regions have been shown to be reciprocally connected in non-human primates (Rempel-Clower and Barbas, 2000). In humans, OFC engages in functional coupling with the medial temporal lobe during memory retrieval (Nyberg et al., 1995; Piefke et al., 2003; Tsukiura and Cabeza, 2008; Anderson et al., 2010; Colgin, 2011). It will be important to further clarify the possibility that OFC engages in association-based memory retrieval during visual perception.

## Summary and Conclusion

We have reviewed evidence that predictions have consequences for conscious perception. When visual input is ambiguous, predictions may help select the contents of awareness, maintaining a coherent interpretation of the environment. Under less demanding conditions, predictions may still influence awareness, allowing percepts to be generated more quickly and with less interference by sensory noise. To support these arguments, we have drawn on observations from a wide variety of domains, including the resolution of binocular rivalry, perception of ambiguous figures, associative learning, and other phenomena. We suggest, however, that all of these studies index the fact that humans are highly adept at extracting consistencies in the world and using this knowledge to generate expectations about the immediate sensory environment. Although different instances of prediction may recruit different cortical regions, predictive mechanisms are likely instantiated as dynamic top-down modulation of sensory cortex by higher sensory and prefrontal areas engaged in comparatively abstract processing. Via this modulation, predictions about the environment generated in higher-level cortical regions can guide perception.

Given that the specific neural processes that give rise to conscious perception remain unclear, it is difficult to conjecture precisely how predictive feedback influences the contents of awareness. However, it is intriguing that a number of prominent theories posit that top-down feedback may play an important role in generating the neural states postulated to account for consciousness (Tononi and Edelman, 1998; Lamme, 2010; Dehaene and Changeux, 2011; Meyer, 2012). Indeed, disrupting top-down processes seems to impair awareness (Pascual-Leone and Walsh, 2001; Ro et al., 2003; Fahrenfort et al., 2007; Dux et al., 2010). Perhaps predictions play not only a modulatory but a driving role in awareness, particularly when other top-down processes such as attention are not engaged. Future research should explore whether an individual’s threshold for visual awareness increases when predictive processes are impaired, such as in depression in which associative processing may be limited (Bar, 2009).

## Conflict of Interest Statement

The authors declare that the research was conducted in the absence of any commercial or financial relationships that could be construed as a potential conflict of interest.

## Footnotes

^1^We use “subjective visual experience,” “conscious perception,” and “visual awareness” interchangeably to refer to the phenomenological experience of seeing, distinct from other forms of consciousness such as self-awareness.

^2^When interpreting the influence of predictions on perception, it is important to consider potential contributions of attention because the two may be easily confounded. However, attention and prediction are dissociable in several ways (Hohwy, 2012; Summerfield and Egner, 2009) and efforts have begun to elucidate the independent contributions of each to perception (Wyart et al., 2012).
